# Multinodular and vacuolating neuronal tumors in epilepsy: dysplasia or neoplasia?

**DOI:** 10.1111/bpa.12555

**Published:** 2017-09-19

**Authors:** Maria Thom, Joan Liu, Anika Bongaarts, Roy J. Reinten, Beatrice Paradiso, Hans Rolf Jäger, Cheryl Reeves, Alyma Somani, Shu An, Derek Marsdon, Andrew McEvoy, Anna Miserocchi, Lewis Thorne, Fay Newman, Sorin Bucur, Mrinalini Honavar, Tom Jacques, Eleonora Aronica

**Affiliations:** ^1^ Departments of Clinical and Experimental Epilepsy and Neuropathology UCL Institute of Neurology and the National Hospital for Neurology and Neurosurgery Queen Square London WCN1BG UK; ^2^ Department of (Neuro)Pathology Academic Medical Center Amsterdam The Netherlands; ^3^ Cardiovascular Pathology Unit, Department of Cardiac Thoracic and Vascular Sciences University of Padua Medical School Padova Italy; ^4^ Neuroradiological Academic Unit, Department of Brain Repair and Rehabilitation UCL Institute of Neurology, Queen Square London WC1N 3BG UK; ^5^ Victor Horsley Department of Neurosurgery National Hospital for Neurology and Neurosurgery Queen Square London WC1N 3BG UK; ^6^ Neurosurgery Department Brighton and Sussex University Hospitals Brighton UK; ^7^ Department of Anatomic Pathology Hospital Pedro Hispano Matosinhos Portugal; ^8^ Neuropathology Department Great Ormond Street Hospital London UK; ^9^ Stichting Epilepsie Instellingen Nederland (SEIN), Heemstede The Netherlands

**Keywords:** Multinodular, vacuolating, neuronal, tumour, epilepsy

## Abstract

Multinodular and vacuolating neuronal tumor (MVNT) is a new pattern of neuronal tumour included in the recently revised WHO 2016 classification of tumors of the CNS. There are 15 reports in the literature to date. They are typically associated with late onset epilepsy and a neoplastic vs. malformative biology has been questioned. We present a series of ten cases and compare their pathological and genetic features to better characterized epilepsy‐associated malformations including focal cortical dysplasia type II (FCDII) and low‐grade epilepsy‐associated tumors (LEAT). Clinical and neuroradiology data were reviewed and a broad immunohistochemistry panel was applied to explore neuronal and glial differentiation, interneuronal populations, mTOR pathway activation and neurodegenerative changes. Next generation sequencing was performed for targeted multi‐gene analysis to identify mutations common to epilepsy lesions including FCDII and LEAT. All of the surgical cases in this series presented with seizures, and were located in the temporal lobe. There was a lack of any progressive changes on serial pre‐operative MRI and a mean age at surgery of 45 years. The vacuolated cells of the lesion expressed mature neuronal markers (neurofilament/SMI32, MAP2, synaptophysin). Prominent labelling of the lesional cells for developmentally regulated proteins (OTX1, TBR1, SOX2, MAP1b, CD34, GFAPδ) and oligodendroglial lineage markers (OLIG2, SMI94) was observed. No mutations were detected in the *mTOR* pathway genes, *BRAF*, *FGFR1 or MYB*. Clinical, pathological and genetic data could indicate that MVNT aligns more with a malformative lesion than a true neoplasm with origin from a progenitor neuro‐glial cell type showing aberrant maturation.

## Introduction

Indolent cortical lesions provoking focal refractory epilepsy often bridge a gap between focal developmental anomalies and low grade tumors. A long history of seizures and a lack of progressive neurology or MRI changes are characteristic of WHO grade I tumors, such as ganglioglioma and dysembryoplastic neuroepithelial tumors (DNT) 57. However, many hamartomatous or developmental ‘overgrowth’ lesions, particularly the mTORopathies 13 or those associated with mutations in the PI3K–AKT signaling pathway 21, are characterized by abnormal cortical architecture with excessive cell size and tumor‐like mass effect, such as tuberous sclerosis.

In 2007, we reported a unique case of a ‘diffuse gangliocytoma’ involving the temporal lobe in a patient with late onset epilepsy which posed such a diagnostic conundrum 45. Pathology examination of an MRI visible lesion disclosed diffuse involvement of the white matter by nodules of vacuolated ganglion cells; malformations as focal cortical dysplasia (FCD), mild malformation of cortical development, nodular heterotopia were diagnoses considered in addition to a neuronal tumour. Recently reports of histologically similar lesions have been termed ‘Multinodular and vacuolating neuronal tumors (MVNT)’ 7, 17, 24 and this entity is now included in the current 2016 WHO classification of CNS tumors as a pattern of gangliocytoma but with ‘uncertain class assignment’ 33. Indeed, it has been commented that these lesions ‘may even be malformative in nature’ and it is acknowledged that further characterization is needed to fully understand their nosological place among CNS neoplasms 34.

In this study, we review the clinical and pathological findings in a series of ten cases of MVNT (including the case in our initial report 45). We explore the cellular differentiation and maturation, genetic abnormalities, and potential epileptogenic mechanisms in an aim to better explore its delineation between cortical malformation or an epilepsy‐associated tumour in the context of long‐term focal epilepsy.

## Methods

### Case selection

Seven cases of MVNT were ascertained from pathology review of all glio‐neuronal tumors and malformations in the Department of Neuropathology and Epilepsy Society Brain and Tissue Bank at UCL. A further three cases of MVNT were included from the Academic Medical Centre (Amsterdam, the Netherlands), Great Ormond Street Hospital for children, (London, UK) and Hospital Pedro Hispano, (Matosinhos, Portugal). There is ethical approval for this study and all surgical patients consented for use of tissue, clinical and MRI data in research. We also included fetal developmental controls (from MRC‐Wellcome Trust Human Developmental Biology Resource HDBR, UCL), conventional gangliogliomas/low grade glioneuronal tumour (WHO grade I), temporal lobe cortex adjacent to hippocampal sclerosis with mild malformation of cortical development type II (increased white matter neurones 6, 32), and FCD type II cases from epilepsy surgical patients, as comparative control groups for immunohistochemistry (Supporting Information Table S1 for details) or genetics studies.

### Immunohistochemistry

Routine H&E and LFB/CV were performed on sections of all cases using the Leica ST5020 Autostainer (Leica, Milton Keynes, UK). From all cases we then selected a representative block and a panel of immunohistochemistry markers (Table 1) was applied on 5 µm formalin‐fixed, paraffin‐embedded brain sections to explore: neuronal differentiation (NeuN, neurofilaments, synaptophysin, MAP2), astroglial differentiation (GFAP and GFAPδ), oligodendroglial lineage and myelination (Olig2, SMI94/myelin basic protein (MBP)) and common glioma mutations (IDH1, ATRX and BRAF V600E). In seven cases with sufficient material available, a more extensive immunohistochemistry panel was applied including cortical layer specific markers (TBR1, TBR2, OTX1, N200, MAP1B), developmental/stem cell markers (CD34, Reelin, PAX6, SOX2, Nestin, DCX, PDGFRβ), interneuronal subsets (calbindin, calretinin, parvalbumin, NPY), chloride co‐transporters (NKCC1, NKCC2), neurodegenerative markers (p62, AT8, APP, mitochondria) and mTOR pathway activation (pS6, Ser 240/244 and pS6, Ser 235/236). Either automated immunohistochemistry was performed (Bond Max Automated Immunostainer (Leica, Milton Keynes, UK)) or it was carried out manually using standard protocols as previously detailed 20 (Table 1).

**Table 1:** List of immunohistochemical markers applied to the study of multinodular vacuolated neuronal tumour (MVNT).

GROUP	Antibody (source)	Epitope/labelling pattern in normal cortex	Pre‐treatment, antibody dilution (min, temperature)
**MATURE NEURONAL MARKERS**	**NeuN** (EMD)	Neuronal nuclear antigen/neuronal nuclei and cytoplasm	ER1, 1:2000 (20, RT)
	**SMI32** (Sternberger)	Neurofilament (non‐phosphorylated 200 kDa protein)/axons and some pyramidal cell bodies	1:500 (20, RT)
	**SMI31** (Sternberger)	Neurofilament (phosphorylated 200 kDa protein)/axons	1:5000 (20, RT)
	**Synatophysin** (DAKO)	Synaptic protein/synaptic vesicles	ER2, 1:100 (20, RT)
	**NFc** (DAKO)	Neurofilament cocktail	1:500 (20, RT)
	**MAP2** (Sigma)	Microtubule‐associated protein/neuronal cytoplasm and processes	H‐3301, 1:1500 (60, RT)
CORTICAL LAYER NEURONAL MARKERS*	**TBR1** (Abcam)	T‐box brain protein/nuclei marker for cortical neurones derived from intermediate progenitor cells (IPC)	H‐3301, 1:400 (ov, 4°C)
	**TBR2** (EMD)	T‐box brain protein/nuclei marker for basal progenitor cells	H‐3301, 1:2500 (ov, 4°C)
	**OTX1** (Abcam)	Orthodenticle homolog 1/expressed in the nuclei of a subset of layer V/VI projection neurones.	H‐3301, 1:100 (ov, 4°C)
	**N200** (Sigma)	Neurofilament 200/projection neurones	H‐3301, 1:3000 (60, RT)
	**MAP1B** (Abcam)	Microtubule‐associated protein 1B/earliest MAP expressed in development; in subset of layer V neurones in fetal cortex.	H‐3301, 1:2500 (ov, 4°C)
**ASTROGLIAL**	**GFAP** (DAKO)	GFAP/astrocytes	ENZ1, 1:2500 (20, RT)
	**GFAP delta** (EMD)	GFAP isoform, developmental regulation/subset of astrocytes, stem cells	H‐3301, 1:5000 (48hrs, 4°C)
**OLIGO‐DENDRO GLIAL/MYELIN**	**OLIG2** (EMD)	Oligodendroglia lineage transcription factor	SC, 1:400 (ov, 4°C)
	**SMI94** (Sternberger)	Myelin basic protein	ENZ1, 1:2000 (20, RT)
STEM CELL/DEVELOP‐MENTAL*	**Reelin** (EMD)	Extracellular matrix protein/expressed in Cajal–Retzius cells in cortical development	H‐3300, 1:6000 (ov, 4°C)
	**PAX6** (Santa Cruz Bio.)	Paired‐box protein; nuclei	H‐3300, 1:100 (ov, 4°C)
	**PDGFR beta** (Abcam)	Platelet derived growth factor receptor beta: NG2/oligodendroglial precursor cell types^†^	H‐3300, 1: 1000 (ov, 4°C)
	**SOX2** (EMD)	Sex‐determining region Y‐box 2/progenitor cells	H‐3300, 1:200 (ov, 4°C)
	**CD34** (DAKO)	Stem cell marker/endothelial cells	1:50 (20, RT)
	**Nestin** (Abcam)	Intermediate filament; developmentally regulated/expressed in stem cells and radial glial	H‐3300, 1:1000 (ov, 4°C)
	**Doublecortin** (Cell Signaling Tech.)	Developmentally regulated neuronal microtubule‐associated protein/neuroblasts	SC, 1:250 (ov, 4°C)
INTER‐NEURONAL*	**Calbindin** (Swant)	Calcium binding protein/interneuronal marker	H‐3300, 1:10,000 (ov, 4°C)
	**Calretinin** (Sigma)	Calcium binding protein/interneuronal marker	H‐3300, 1:3000 (ov, 4°C)
	**Parvalbumin** (Swant)	Calcium binding protein/interneuronal marker	H‐3300, 1:5000 (ov, 4°C)
	**Neuropeptide Y** (Sigma)	Neuropeptide/GABAergic neurones	H‐3300, 1:4000 (ov, 4°C)
	**KCC2** (Autogen Bioclear)	K+/Cl− Cotransporter/GABAergic neurones	1:600 (ov, 4°C)
	**KCC1** (Gift) **(NKCC1)**	Na+/K+/Cl− Cotransporter isoform/GABAergic neurones	H‐3301, 1:500 (60, RT)
NEURO‐DEGENERATIVE*	**AT8** (Innogenetics)	Phosphorylated microtubule‐associated tau protein	ER1, 1:1200 (20, RT)
	**APP** (EMD)	Amyloid precursor protein	ER1, 1:800 (20, RT)
	**p62** (BD)	Sequestosome‐1/targets specific cargoes for autophagy	ER2, 1:100 (20, RT)
	**Mitochondria** (Abcam)	Anti‐mitochondrial antibody	ER2, 1:200 (20, RT)
**MTOR PATHWAY ACTIVATION**	**pS6 Ser240/244** (Cell Signaling Tech.)	Phosphorylated‐S6 ribosomal protein at ser 240/244	H‐3301, 1:1000 (ov, 4°C)
	**pS6 Ser 235/236** (Cell Signaling Tech.)	Phosphorylated‐S6 ribosomal protein at serine 235/236	H‐3301, 1:250 (ov, 4°C)
**CELL CYCLE AND TUMOUR MUTATION MARKERS**	**MCM2** (BD)	Mini chromosome maintenance protein/cells licensed for replication	H‐3301, 1:900 (ov, 4°C)
	**Ki67** (DAKO)	Nuclear protein/proliferating cells	ER2, 1:200 (20, RT)
	**BRAF V600E** (Spring Bioscience)	V600E mutation to Serine/threonine‐protein kinase B‐raf/upregulated in benign and malignant human tumours	ER2, 1:50 (20, RT)
	**IDH1** (Dianova)	Isocitrate Dehydrogenase 1 mutation R132H low grade and secondary high grade gliomas	ER2, 1:64 (20, RT)
	**ATRX** (Sigma)	Alpha thalassemia/mental retardation syndrome x‐linked (is SNF2 family of helicase and ATPases)/gliomas	ER2, 1:500 (20, RT)

For markers in the groups of antibodies indicated with * between 4 and 8 cases with MVNT were studied with each marker due to limited availability of sections; for remaining cases, all markers were examined in all cases.

^†^PDGFRbeta in this series was used to label pericytes as well as small multipolar NG2‐like glial cells as previously reported 18, 55; reliable labelling of NG‐2 cells with NG2 or PDGFRalpha was not achieved in these cases. Antigen retrieval buffers (buffers used in auto‐immunostainer is in bold): **ENZ1**, Bond enzyme concentrate and diluent (Leica, Milton Keynes, UK); **ER1**, Bond citrate‐based buffer (Leica, Milton Keynes, UK); **ER2** Bond EDTA‐based buffer (Leica, Milton Keynes, UK); H‐3301 Vector's Tris‐based buffer pH9.0 (Vector Lab, Peterborough, UK); H‐3300 Vector's citrate‐based buffer pH6.0 (Vector Lab, Peterborough, UK); SC Sodium Citrate buffer pH6. RT, room temperature; OV, overnight. Suppliers: EMD Millipore, Watford, UK; Sternberger, MD, USA; DAKO, Cambridgeshire, UK; Sigma Aldrich, Dorset, UK; Abcam, Cambridge, UK; Santa Cruz Bio., Heidelberg, Germany; Cell Signaling Tech., Boston, MA, USA; Swant, Marly, Switzerland; Autogen Bioclear Ltd, Wiltshire, UK; BD Transduction Lab., Oxford, UK; Spring Bioscience, CA, USA; Dianova, Hamburg, Germany.

### Next generation sequencing

Next generation sequencing (NGS) of MVNT was carried out in eight of the nine surgical samples (Case 7 not sequenced due to lack of availability of frozen tissue and the post mortem Case 4 failed sequencing due to low quality of DNA). Tumour tissue was manually micro dissected from 10 μm tissue sections. DNA was extracted using the BiOstic FFPE Tissue DNA Isolation kit (MO BIO, Carlsbad, CA, USA) according to the manufacturer's instructions. NGS was performed using a customized Ion AmpliSeq^™^ Neurology Panel (ThermoFisher Scientific, Waltham, MA, USA) for targeted multi‐gene amplification. This panel consists of the following genes; AKT1, AKT3, ATRX, BRAF, CDK6, CIC, CTNNB1, DDX3X, DEPDC5, EZH2, FGFR1, FUBP1, H3F3A, HIST1H3b, HIST1H3c, IDH1, IDH2, KDM6A, mTOR, MYB, MYBL1, NPRL2, NPRL3, PIK3CA, PIK3R1, PIK3R2, PTCH1, PTEN, SMARCA4, SMARCB1, SMO, SUFU, TP53. Libraries were prepared using the Ion AmpliSeq Library Kit 2.0. The Ion PGM Hi‐Q Kit and Ion Chef Instrument were used for emulsion PCR and template preparation. The Ion PGM Hi‐Q sequencing Kit with the Ion 318 V2 Chip and Personal Genome Machine were used as sequencing platforms. DNA input was up to 20 ng, which was measured by the Qubit 3.0 Fluorometer. Up to 20 specimens were barcoded using the IonXpress Barcode Adapters for each Ion 318 V2 Chip. A mean coverage of 1500× per amplicon was established, and the data were analyzed using JSI SeqNext (JSI Medisys, Ettenheim, Germany). Of note, the selected sequencing panel was developed to identify most mutations in the genes studied. However, not all exons of each gene were analyzed, thus there is a risk of undetected mutations in tumour suppressor genes. Gene duplications were not detected, and the sequence analysis of MVNT was compared to 22 samples with FCD type II and 8 glioneuronal tumors.

### Clinical data and MRI

The clinical notes from each case were reviewed for presenting neurological symptoms and updated post‐operative information. MRI had been carried out in different centers using different MRI sequences and protocols. All available pre‐operative MRI scans of surgical cases (Cases 1, 2, 5, 6, 8 and reports in the remainder) were reviewed by a neuroradiologist (HRJ) in addition to post‐operative MRI findings of any disease progression/recurrence.

## Results

Of the 10 cases with MVNT, nine were surgical resections carried out for treatment of epilepsy, and a further case was identified at post‐mortem as an incidental finding (Table 2). One surgical case was previously reported as an unusual gangliocytoma (Case 1) 45, and two further surgical cases were initially diagnosed as a gangliocytoma and a demyelinating condition (Cases 2 and 3) following histopathological assessment; the PM case was diagnosed as subcortical grey matter heterotopia (Case 4). The remaining six surgical cases (all reported since 2014) were recognized as MVNT.

**Table 2:** Clinical details of cases in current series, location, salient MRI features and outcome following surgery. Case 1 was previously reported (Ratilal *et al*., 2007). PM = post mortem; AD = Alzheimer's disease; NA = not applicable; PS = partial seizures; GS = generalized seizures.

Case	Presenting symptoms	Age onset of seizures (years)	Age at surgery (PM)/Gender	Type of seizures	Localization structures involved	Type of surgical resection	Any other relevant neurology	Outcome following surgery. Seizure control/tumour recurrence [period of follow up]
1	Seizures	39	59/F	PS, GS	Left temporal lobe, parahippocampal gyrus, hippocampus and amygdala	Left temporal lobectomy, hippocampectomy and resection of amygdala	Declining verbal memory post operatively. Depression	Continued seizures (1–3 nocturnal seizure per week)/no recurrence of tumour [9 years]
2	Seizures/temporal lobe epilepsy	‘epilepsy many years’	32/F	PS	Right temporal lobe, parahippocampal gyrus, hippocampus and amygdala	Right temporal lobectomy, hippocampectomy and resection of amygdala	Impaired non‐verbal learning. Migraine	Seizure‐free/no recurrence [6 years]
3	Seizures	10	27/F	PS, GS	Left amygdala and hippocampus	Left temporal lobectomy, hippocampectomy and resection of amygdala	Psychosis following temporal lobectomy	Seizure‐free/no recurrence on MRI [14 years]
4	Breathing difficulties	NA	62/M	No history of epilepsy	Right occipital lobe	Post mortem: whole brain	Braak stage V AD diagnosed at PM Cause of death mesothelioma	NA
5	Visual symptoms/seizures	65	67/F	PS	Right posterior parietal‐temporal lobe lesion	Lesionectomy	None	No neurological deficit and seizure free/lesion stable [2 years]
6	Seizures/speech arrest	47	48/F	PS, GS	Left middle temporal gyrus	Lesionectomy/awake craniotomy	None	Seizure‐free/residual tumour but no progression on MRI [2 years]
7	Seizures		6/M		Left temporal	Left temporal neocortex, hippocampectomy		Seizure‐free/residual tumour on MRI but no progression [17 months]
8	Seizures	21	54/M	PS,GS	Right temporal lobe, parahippocampal gyrus, fusiform gyrus and hippocampus	Temporal lobe, hippocampectomy	Postictal psychosis/post operative facial weakness	Seizure‐free/no progression [10 months]
9	Epilepsy		41/F		Right mesial temporal lesion	Temporal lobe resection		Partial resection but no progression on MRI
10	Epilepsy		55/M	CPS	Temporal lobe and temporal pole	Temporal lobe resection		Recent surgery, Limited follow up data

### Clinical features

Six of the ten cases were females. The mean age at seizure onset was 29.7 years based on information available (Table 2) with a mean age at surgery of 45 years (range 6–67 years). The single post‐mortem case was a 62‐year‐old male without a clinical diagnosis of epilepsy. In five surgical cases, the lesions primarily involved the temporal lobe, and mesial structures (hippocampus, amygdala or parahippocampal gyrus); the post‐mortem lesion involved the occipital lobe. Clinical outcomes were available up to 14 years post‐operatively, however four cases were recently operated with less than 2 years of follow‐up. Only one patient did not become seizure‐free post‐operatively, and no case has documented lesion progression on MRI, even in cases with partial resections (Table 2).

### Neuroimaging features

**Figure 1:**
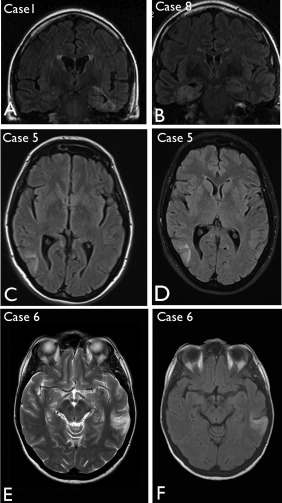
Neuroimaging features of multinodular and vacuolating neuronal tumour. **A**. Case 1 (as reported in the original case report^45^) showing marked signal change in the left temporal lobe, parahippocampal gyrus and hippocampus on coronal FLAIR but with minimal mass effect; little change was noted over 8 years. **B**. Case 8. Abnormality was observed in the right mesial temporal region with hyperintensity on coronal FLAIR sequence. **C**. Case 5. Initial FLAIR MRI sequences at presentation highlighted a lesion in the right posterior temporal lobe as a diffuse cortical abnormality, which did not show significant growth on MRI, as shown in (**D**) at 21 months following the initial scan. **E**. Case 6 shown with T2 and (**F**) FLAIR sequences, highlighting a hyperintense abnormality and the cortical, white matter interface in the left temporal lobe.

Salient MRI features common to all cases included poorly defined regions of signal abnormality in the temporal lobe, typically hypo‐intense on T1 with hyperintensity on T2 and FLAIR sequences, involving the cortex and straddling the subcortical region with more clear involvement of the adjacent white matter in some cases (Figure 1). A vague, corrugated contour was apparent in some but without clear nodularity or cystic change. Although the extent of the abnormality varied between cases, there was no or minimal mass effect associated with these lesions. There was no enhancement or restricted diffusion where investigated. Clear involvement of the hippocampus was apparent in Cases 1, 2, 8 and 9 (Figure 1A,B). There were no progressive changes on serial images in the pre‐operative period, in one case with six interval MRI studies (Case 5, Figure 1C,D) and over review periods of up to 8 years (Case 1, Figure 1A). Low grade glioma was considered the likely diagnosis or, less likely cortical dysplasia, based on imaging features. None of the lesions showed progressive changes during the post‐operative period (Table 2).

### Neuropathology findings

**Figure 2:**
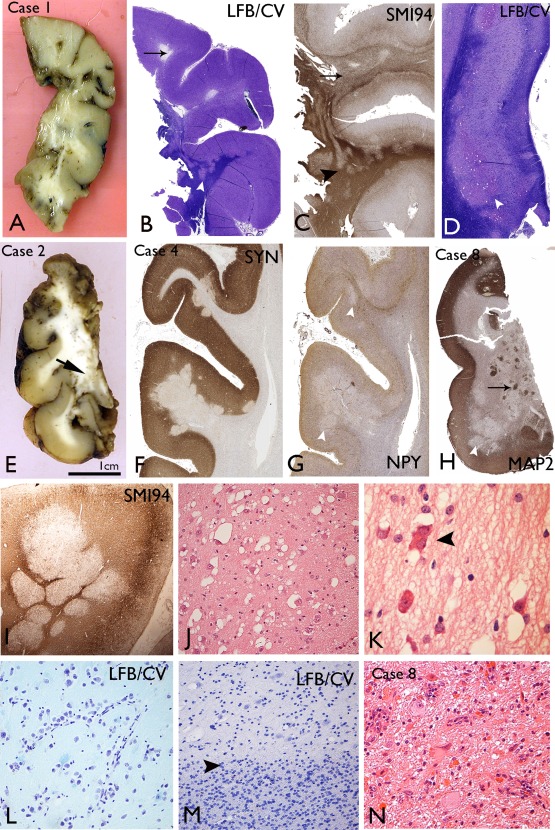
Macroscopic and low power histological features of multinodular and vacuolating neuronal tumours (MVNT). **A**. Macroscopic features of the temporal lobe specimen from Case 1 show areas of breakdown/cavitation in the gyral cores and focal translucency of the white matter. **B**. Luxol fast blue/cresyl violet stained section from Case 1 confirms lack of myelin in some gyral cores (arrow) and pale hypomyelinated nodules at the cortical white matter junction extending into the white matter (arrowhead). **C**. SMI94/myelin basic protein confirms diffuse regions of poor myelination (arrow) and nodular like patterns (arrowhead) in the white matter in Case 1. **D**. Abnormal nodular islands of cells in the subiculum in Case 1, present on both sides of the pyramidal cell layer, although predominantly in the subcortical region (arrowhead). **E**. Macroscopic appearance of fixed 5 mm sections of the temporal lobe in Case 2 shows islands of grey tissue in the white matter of the inferior medial part of the specimen with an overlying normal‐appearing cortex. **F**. Synaptophysin labelling in Case 4 highlights the nodules encroaching on the deep cortical layers with reduced labelling compared to the adjacent cortex. **G**. Neuropeptide Y in Case 4 at low power shows reduction of labelling within the cortical nodules compared to adjacent cortex (arrowheads). **H**. MAP2 staining in Case 8 in the temporal cortex shows variable patterns with reduced MAP2 labelling in some nodules (arrowhead) compared to others (arrows). **I**. Myelin basic protein staining (SMI94) in Case 4 highlights abutting, myelin‐poor nodules. **J**. The abnormal white matter regions are populated by single scattered cells with a neuronal/ganglion cell appearance and prominent vacuolation of the cytoplasm or vacuoles surrounding the cells. **K**. Occasional binucleated cells were seen (arrowhead) and cells with more eosinophilic cytoplasm were observed in MVNT after H&E staining. **L**. In some cases alignment of the atypical cells along vessels was noted in the nodules (Case 4). **M**. The border (arrowhead) between a nodule in MVNT(top half of figure) and the white matter (lower half of figure) on cresyl violet stain gives the impression of overall reduced cellularity, particularly for small oligodendroglial‐like cells in the nodules, compared to the adjacent white matter. **N**. In Case 8, a focal area in the mesial temporal lobe showed more typical appearances of a ganglioglioma, with dysplastic neurones and eosinophilic granular bodies. Bar = 1cm for macroscopic images in A and E; = 3mm for B–D, F–H; =0.5mm for I; = 100 microns for J, L–N and =50 microns for K (approximate based on original magnifications).

Macroscopic abnormalities noted in fixed, resected lobectomy specimens included focal translucency or cavitation of the subcortical white matter in the gyral cores of the temporal lobe white matter (Figure 2A) or with plaque‐like nodules or islands of grey discoloration (Figure 2E). In the post‐mortem specimen, nodules of grey matter were noted in the subcortical white matter of the right occipital lobe only.

Histological examination at low magnification in larger resections confirmed islands or nodules, predominantly located in the subcortical white matter or ‘sitting’ in the deeper cortical layers. Coalescing or abutting nodular islands (Figure 2I) as well as areas of more diffuse subcortical white matter involvement (Figure 2C) were observed. The architecture and extent of the white matter component at low power was best appreciated by reduced myelin on myelin stained sections (LFB/CV or SMI94/MPB) (Figure 2B–D,I) and for the cortical component, reduced synaptophysin (Figure 2F). In regions bearing cortical nodules, the laminar cyto‐architecture and myelo‐architecture of the cortex was otherwise undisturbed. The nodules were composed of large cells with overall gangliocytic morphology, prominent nucleoli with frequent cytoplasmic vacuolation or peri‐cellular vacuolation (Figure 2J). Cells with more eosinophilic cytoplasm and rarer bi‐nucleate cells were present (Figure 2K). There was minimal clustering and no orientation of vacuolated cells (VC) within nodules but arrangements alongside vessels were noted in one case (Figure 2L). In addition to the predominant VC, smaller oligo‐like glial cells were intermingled, but without satellitosis. The impression in some nodules was of diminished cellularity compared to adjacent white matter (Figure 2M) although in other cases (Case 7) regions of diffuse hypercellularity were noted. Mitotic activity and significant pleomorphism was lacking. Rosenthal fibers or calcification were not present and prominent perivascular infiltrates were noted in four cases.

In five cases, the involvement of the hippocampus was present, with islands of VC in the white matter underlying the subiculum (Figure 2D), CA1 and as well as parahippocampal gyrus white matter and amygdala. In Case 8, the mesial temporal component showed cytology more typical for ganglioglioma with dysmorphic ganglion cells, eosinophilic granular bodies (EGBs) (Figure 2N) and nodules of spindled astroglial cells, even though the temporal lobe component had the typical appearances of MVNT (Figure 2H); this suggested a tumour with hybrid features. Hippocampal sclerosis was also noted in three cases.

### Neuronal differentiation

**Figure 3:**
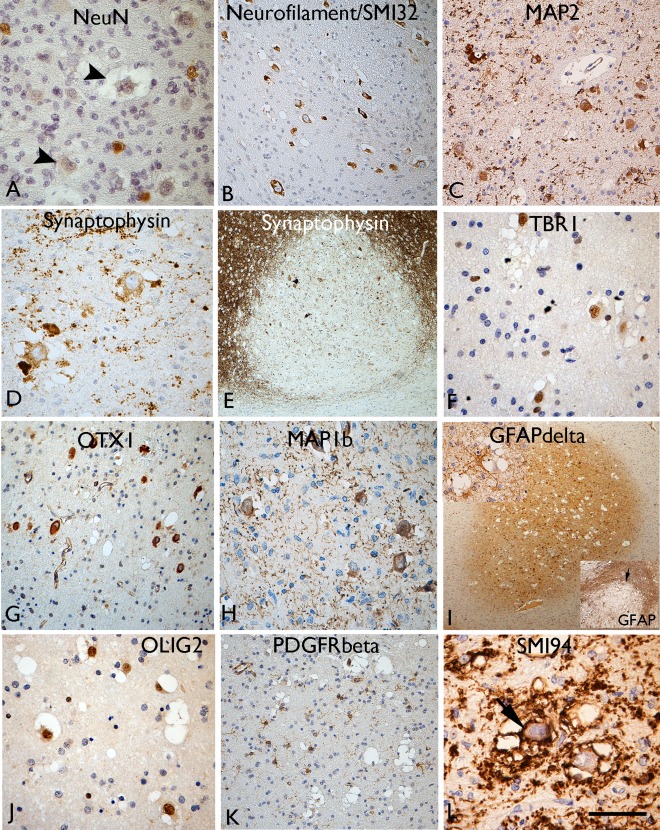
Neuronal and glial marker expression in vacuolated cells (VC). **A**. NeuN staining showed labelling of normal interstitial neurons in the white matter, but the vacuolated cells (VC) were typically weak or more often negative (arrowheads). Labelling of VC with (**B**) neurofilament, (**C**) MAP2, (**D**) synaptophysin is shown. **E**. Overall, reduced labelling with synaptophysin was observed in the cortical nodules. **F**. TBR1 neuronal lineage marker shows nuclear labelling of t VC and strong cytoplasmic labelling noted with (**G**) OTX and (**H**) MAP1b. **I**. GFAPδ highlighted small glial cells primarily in the nodules at low power; inset (top) multipolar GFAPδ cells in proximity to VC), inset (bottom) GFAP conventional isoform shows opposite pattern with more extensive labelling of the gliosis around the lesions. **J**. Nuclear labelling of VC with OLIG2 whereas (**K**) PDGFRbeta highlighted small multipolar cells in the nodules but not the VC. **L**. SMI94 for myelin basic protein, as well as demonstrating the diminished myelination in the white matter nodules (see also Figure 2), also showed membranous labelling of the VC (arrow).Bar = 80 microns (A, C, D, F–H, J) = 40 microns (I) and 140 microns (B, K) and 200 microns (E and I) (approximate, based on original magnifications).

Immunolabelling using the mature neuronal marker, NeuN, was weak or negative (Figure 3A) in VC, in keeping with previous reports 7, 24 but we confirmed consistent, strong expression for non‐phosphorylated neurofilament (SMI32) in VC in all cases (Figure 3B). Other neurofilament antibodies (Table 1), including phosphorylated neurofilament (SMI31), neurofilament cocktail and N200 labelled only occasional axons traversing through nodules but VC were mainly negative. MAP2 highlighted VC and dendrites (Figure 2H and 3C) and a proportion of the small multipolar cells in nodules. In Cases 3 and 4, the MAP2 labelling was weaker in VC compared to other overlying cortical neurones. Despite an overall reduction of synaptophysin expression in the nodules (Figure 3E), imparting a ‘moth‐eaten’ appearance to the cortical‐white matter border at low magnification (Figure 2F); a proportion of VC showed weak cytoplasmic labelling in all cases (Figure 3D). In Case 8 with a focal ganglioglioma component, stronger synaptophysin labelling of atypical ganglion cells was noted compared to the VC.

### Cortical layer differentiation markers

Application of cortical layer and neuronal linage markers revealed variable labelling of VC for TBR1; a proportion of VC in four of eight MNVN showed strong nuclear positivity (Figure 3F) while in other cases, VC were weak or negative. The overlying cortex showed TBR1 nuclear labelling of neuronal cells in all cortical layers. In Case 8 with the ganglioglioma component, dysplastic neurons were negative with TBR1. There was no labelling of VC for TBR2 in any case. Intense cytoplasmic labelling of VC for OTX1 was noted in all cases apart from the post‐mortem case (Figure 3G); in comparison the overlying cortex and normal white matter was negative with this marker in keeping with previous reports 20. OTX1, therefore, appeared a specific marker to distinguish VC from normal interstitial neurones in the white matter as well as delineating the extent of the MVNT. With MAP1b, we also observed intense cytoplasmic labelling of the VC and their dendritic like processes in all cases (Figure 3H) in addition to labelling of pyramidal cells of normal morphology in layers II, III and V of the overlying cortex.

### Glial lineage markers

GFAP accentuated variable proportions of small, multipolar astroglial cells and processes in the lesion typically merging with a denser gliosis in the surrounding white matter (Figure 3I, bottom inset). In Case 8 with a ganglioglioma component, a more prominent GFAP‐positive glial tumour component was confirmed. GFAPδ showed restricted expression in MVNT, localizing to the nodules and diffuse components at low magnification (Figure 3I), labelling small multipolar glial cells in proximity to VC and sometimes enveloping them (Figure 3I, top inset). In keeping with previous reports 7, 24 VC showed strong nuclear labelling for OLIG2 (Figure 3J); a proportion of the smaller glial cells associated with the lesion were OLIG2‐positive. NG2 and PDGFRα immunomarkers did not show any consistent labelling of the surgical cases or post‐mortem tissue. However PDGFRβ, which labels both pericytes and NG2/oligodendroglial progenitor cells 18, 55, small multipolar cells within in the nodules away from vessels as well as in adjacent tissue were noted (Figure 3K); the large VC were negative with PDGFRβ. MBP (SMI94) showed striking abnormalities, correlating with overall reduction of myelinated fibers in the lesion as described above (Figure 2C,I). In addition, cytoplasmic membranous or granular labelling of VC was noted for MBP (Figure 3L) and the rims of empty vacuoles also highlighted.

### Neurodevelopmental and interneuronal markers

**Figure 4:**
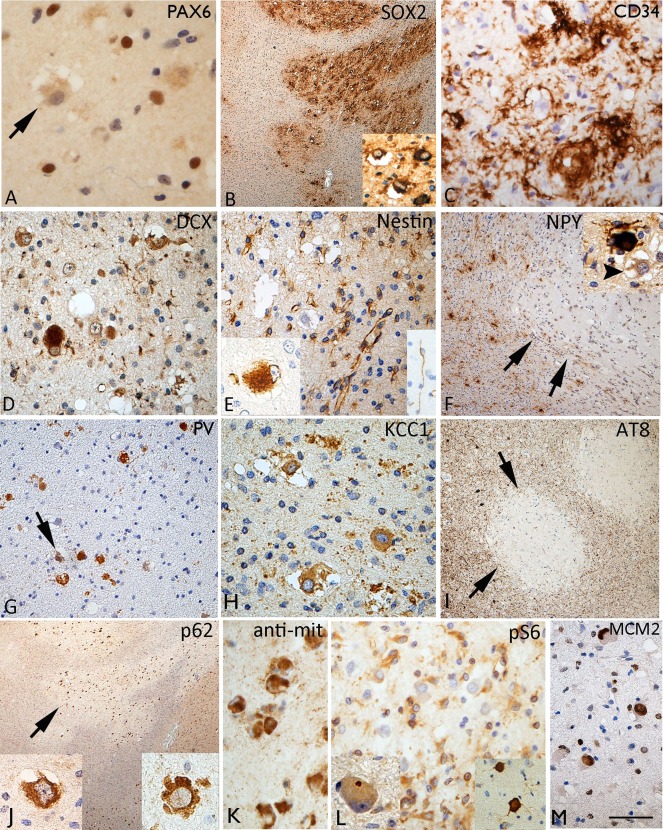
Immature and interneuronal markers in multinodular and vacuolating neuronal tumour. **A**. PAX6 was negative in vacuolated cells (VC) but labelled nuclei of small cells within the lesion. **B**. SOX2 showed strong labelling of the nodules and VC highlighting the regions of involvement at low magnification (**C**) CD34 showed variation in the staining between cases, but as shown here in Case 5, prominent multipolar cells and processes were evident in the regions with VC. **D**. Occasional doublecortin positive (DCX) VC were seen. **E**. VC were mainly nestin negative but occasional positive cells were seen (inset left). Small nestin expressing cells were noted in the lesion and occasional bipolar cell (inset right). **F**. NPY showed reduced labelling in nodules of one MVNT (Case 4), arrows showing the edge of the nodule; NPY staining labelled scattered interneurons in the lesion of normal morphology but the VC were negative (arrowed in inset). **G**. Case 3 showing strong labelling of VC for parvalbumin. **H**. Cationic chloride transporter ((N/KCC1) with distinct cytoplasmic labelling of the VC. **I**. AT8 labelling for phosphorylated tau in Case 4 with Alzheimer's disease pathology, and dense cortical tau in tangles and threads showed a marked sparing of the VC and nodules on MVNT for tau accumulation (arrows). **J**. VC were strongly positive with p62 and (**K**) anti‐mitochondrial antibodies. **L**. VC were not positive with both pS6 markers pS6 Ser240/244 (shown here) and pS6 Ser 235/236, apart from occasional dot‐like positivity in the cytoplasm of uncertain significance (insert left); in contrast the overlying cortex (insert left) showed strong scattered neuronal positivity. **M**. A high proportion of VC were MCM2 nuclear positive. Bar = 50 microns (A, D, E, G, H, K–M) = 20 microns (B, F, I, J) (approximate based on original magnifications).

VC were largely negative for reelin, and cells with the morphology of Cajal–Retzius cells were not seen within the nodules, although they were present in the overlying cortex. VC were also mainly negative with PAX6 which showed nuclear labelling of a proportion small cells in the vicinity of the nodules (Figure 4A). Consistent cytoplasmic or membranous labelling of VC in MVNT with SOX2 was seen in all cases studied, demarcating the nodular and serpiginous islands of the lesion at low magnification (Figure 4B). CD34‐positive multipolar cells were present in all cases, ranging from very occasional cells (Case 2) to extensive labelling (in Cases 7, 8) (Figure 4C). With DCX and nestin, only occasional VC were positive (Figure 4D,E), both markers showed more prominent labelling of small cells associated within the lesion. Nestin also highlighted a population of bipolar cells with long processes in some nodules (Figure 4E; inset). Calbindin, calretinin, parvalbumin and NPY (Figure 4F) showed a normal morphology and distribution of interneurons in adjacent cortex and white matter. VC were generally negative with these markers, apart from one case (Case 3) which showed PV expression in VC (Figure 4G). Interneurons of normal morphology and axonal networks were also intermingled in the MVNT, although there was an impression for reduced numbers compared to adjacent tissue in some cases (Figure 2G and 4F). KCC1 showed strong cytoplasmic labelling of VC (Figure 4H) but labelling with KCC2 was less distinct.

### Neurodegenerative, mTOR pathway and cell cycle markers

In none of the surgical cases were tangles noted in VC, and AT8 for phosphorylated‐tau was virtually negative. In the post‐mortem case with a neuropathology diagnosis of AD, the cortical nodules of the MVNT, in fact, were strikingly spared from tau accumulation compared to the adjacent cortex (Figure 4I) suggesting late acquisition of degenerative changes. VC showed cytoplasmic labelling with APP and intense labelling for p62 (Figure 4J) and anti‐mitochondrial antibodies (Figure 4K) compared to adjacent cortical and white matter neurones. With mTOR pathway antibodies, anti‐pS6 (ser235/236 and ser240/244), minimal labelling of VC was observed across all cases; occasional dot‐like positivity in the cytoplasm was noted in some VC, in contrast to the intense labelling of scattered pyramidal cells in the overlying cortex (Figure 4L; insets) and the dysmorphic neurons of the ganglioglioma component of Case 8. However, strong cytoplasmic labelling for pS6 antibodies of small glial‐like cells associated with the lesional nodules was noted (Figure 4L). A high proportion of VC showed nuclear labelling with MCM2 (Figure 4M) in contrast to Ki67 labelling which was virtually absent in VC, but noted mainly in small cells and inflammatory cells (overall <5%). In all MVNT cases, immunohistochemistry for mutant IDH1, ATRX and BRAF V600E mutations were negative.

### Comparison of MVNT to controls groups

In ganglioglioma controls, Tbr1 nuclear labelling of dysmorphic neurons was noted in one case (Supporting Information Figure 1A); a variable proportion also showed cytoplasmic OTX1 positive labelling (Supporting Information Figure 1B) but more consistent labelling was observed with SOX2 (Supporting Information Figure 1C). Little evidence of nuclear labelling of gangliogliomas for OLIG2 was seen (Supporting Information Figure 1D) but strong labelling with calbindin as previously noted (Supporting Information Figure 1E) 62 and also with p62 as previously reported 43. Dysmorphic cells in ganglioglioma infrequently labelled with MCM2 in contrast to MVNT (Supporting Information Figure 1F). In temporal lobe white matter with mild MCD type II, strong Tbr1 labelling of interstitial white matter neurons was noted (Supporting Information Figure 1G) but not OTX1 (Supporting Information Figure 1H), and rare positive neurones were observed with SOX2 (Supporting Information Figure 1I) or Mapb1b; OLIG2 was restricted to mature‐appearing oligodendroglia (Supporting Information Figure 1J). pS6 expression in TLE white matter showed occasional labelling of white matter neurons and small glial cells (Supporting Information Figure 1K) but no labelling with KCC1 (Supporting Information Figure 1L). In fetal developmental controls, strong labelling, primarily in the periventricular germinal matrix, was confirmed with Tbr2, OTX1 and SOX2 (Supporting Information Figure 1M–O).

### Molecular genetics

Of the 33 genes studied, single mutations in *SUFU* was identified in Case 2, and in EZH2 in Case 8 (Table 3). No mutations in *MTOR*, *DEPDC5*, *FGFR1*, *MYB* or *BRAF* were identified in any MVNT. Recurring synonymous SNPs in *DEPDC5*, *SMO* and *TP53* were present in all surgical MVNT tested, with less frequent synonymous SNPs occurring in some MVNT only (Table 3 and Supporting Information Table S2). We compared the MVNT to FCD type II (22 samples) and 8 glioneuronal tumors; SUFU mutation was present in one FCD type II case but not in other glioneuronal tumors. Identical *DEPDC5* and *SMO* E3 SNP also occurred in all FCD type II samples and the majority of glioneuronal tumors, while the other common SNPs did not occur in all FCD or glioneuronal tumors. Of note, a *NPRL3* variant was found in 5/8 MVNT and 90% of FCD but none of the glioneuronal tumors. The genetic and immunohistochemistry findings in MVNT are summarized and compared to findings in controls as well as published data on related tumors and malformations associated with long‐term epilepsy in Supporting Information Table S3.

**Table 3:** Mutations and recurring, common single nucleotide polymorphisms identified by next generation sequencing in a study of nine multinodular vacuolating neuronal tumours.

Gene	Exon	Position (hg19)/(hg38)	Nucleic change	AA change	Nucleic name	SNP database no	Mutation effect	Total cases MNVT [case number]	Positive FCDII cases	Positive mixed low‐grade glioneuronal tumours
SUFU	8	108 (1018) [chr10: 104359297] [chr10: 102599540]	G→T	A→S (340)	c.1018G>T	rs34135067	Missense mutation of unknown significance	1 [2]	1/22	0/8
EZH2	6	69 (553) [chr7:g.148525904 (hg19)]	G → C	D → H (185)	c.553G>C	rs2302427 COSM3762469 COSM3762470	Missense mutation of unknown significance	1 [8]	0/22	0/8
DEPDC5	9	−43 [chr22:32179850] [chr22: 31783864]	G→C	Intron	c.484–43G>C	rs138286	Intron	8 [all cases tested; 1,2,3,5,6,8,9,10]	22/22	6/8
NPRL3	11	−102 [chr16:142825] [chr16:92827]	A→G	Intron	c.1032–102A>G	rs2541618	Intron	5 [2,3,5,6,9]	20/22	0/8
CIC	20	74 (4533) [chr19: 42799049] [chr19: 42294897]	C→T	I→I (1511)	c.4533C>T	rs1052023 COSM3756833	Silent	6 [1,3,6,8,9,10]	14/22	4/8
PIK3CA	6	−17 [chr3:178922274] [chr3: 179204486]	C→A	Intron	c.1060–17C>A	rs2699896	Intron	7 [2,3,5,6,8,9,10]	19/22	6/8
SMO	3	−26 [chr7:128845018] [chr7:129205177]	C→T	Intron	c.538–26C>T	rs2703091	Intron	8 [all cases tested; 1,2,3,5,6,8,9,10]	22/22	8/8
	6	24 (1164) [chr7:128846328] [chr7:129206487]	G→C	G → G (388)	c.1164G>C	rs2228617 COSM4004294	Silent	8 [all cases tested; 1,2,3,5,6,8,9,10]	21/22	7/8
TP53	4	119 (215) [chr17:7579472] [chr17:7676154]	C→G	P→R (72)	c.215C>G	rs1042522 COSM3766190–93 COSM250061	Polymorphism	8 [all cases tested; 1,2,3,5,6,8,9,10]	20/22	7/8

## Discussion

In 2014, Huse *et al*., described ten cases of a novel low‐grade neuronal lesion, mainly associated with epilepsy and coined the term ‘multinodular and vacuolating neuronal tumour’ 24. Subsequently, five more cases have been reported 7, 17, 39, 68, and this new entity was added to the list of patterns of CNS neoplasms in the recently revised 2016 WHO classification 34. We present the pathology in a further ten cases, including a case previously reported in 2007 as an unusual vacuolated diffuse gangliocytoma 45. Our findings confirm their indolent behavior, and predominant involvement of the temporal lobe presenting with late onset of epilepsy, typically in adulthood. We have extended investigations into their neoplastic vs. malformative nature, cell lineage and potential mechanisms of epileptogenesis through the application of immunohistochemistry and NGS. It remains possible that MVNT align more with developmental anomalies than true neoplasms. Although these lesions are rare, it is probable they have been previously unrecognized, being misdiagnosed as demyelinating conditions 7 or other more common lesions encountered in epilepsy series (as listed in Supporting Information Table S3) with which they share histological similarities.

### Neoplasia or malformation

MVNT has been grouped as a pattern of gangliocytoma in the revised 2016 WHO classification of CNS tumors. Features in common with other low‐grade epilepsy‐associated tumors (LEATs) 57 include a predilection for the temporal lobe, MRI characteristics 39, 68, frequent CD34‐positivity, and a mixed neuronal and glial composition. We include the first report of a case with mixed features of both MVNT and conventional ganglioglioma in support of a potential common origin, and we identified two cases with novel mutations in SUFU and EZH2, although these mutations are of uncertain significance.

As previously noted 24, 45, some features of MVNT align more with a malformation of cortical development or dysplasia than a neoplasm, supported by evidence in this current study. This includes (i) the lack of any growth on serial MRI or reported regrowth, even following incomplete resection, (ii) no overt increase in cellularity or conspicuous mitotic activity, (iii) lack of expansive or infiltrative growth patterns with nodules ‘sitting’ in an undisturbed laminar cortex, (iv) comparable localizations in deep cortex/subcortical region in reported cases, (v) retained expression of immature, developmentally regulated proteins as SOX2, TBR1, OTX1, KCC1 and GFAP∂, (vi) absence of any of the known genetic abnormalities of LEATs following NGS, including *BRAF* V600E, *MYB* and *FGFR1* mutations 15, 25, 42, 44, 47, 50 and (vii) NGS in the present study showing recurring synonymous SNPs in SMO and DEPDC5 in common with cortical dysplasia (FCD IIB) and *NPRL3* SNPs noted frequently in MNVT and FCD but not other glioneuronal tumors. Copy number variations in *SMO* a receptor in the Shh pathway have been recently shown in hypothalamic hamartoma associated with gelastic seizures 22 and *DEPDC5* and *NPRL3* mutations reported in FCDII 5, 56. Further histological features in common between MVNT and FCD IIB include the striking hypomyelination of involved white matter which may relate to deficiencies in oligodendroglial lineages 46, 51, 55, 70. Also the distinctive MCM2 positivity of VC is reminiscent of balloon cells in FCD 61. MCM2 labelling of balloon cells is considered not to reflect neoplastic cell proliferation, but cell cycle arrest in a pathological progenitor cell type 60, 69.

### Lineage and maturity of vacuolating cells

Previous reports have emphasized the preferential expression of immature markers, including HuC/HuD, compared to mature neuronal markers as NeuN in the lesional cells 7, 17, 28, 39, 68. Our study supported evidence for mature neuronal differentiation of VC with labelling for some anti‐neurofilament antibodies (particularly SMI32) although NeuN was typically weak or negative. Expression of DCX, a microtubule‐associated protein (MAP) expressed in migrating neuronal precursors was previously reported in a MVNT 39 but not consistently expressed in our series. We noted however more robust expression with MAP1b, which is highly developmentally regulated but normally retained in neurogenic areas in the adult brain or regions undergoing structural plasticity 66. Strong expression of nodules for alpha‐internexin, an intermediate neurofilament expressed in neuroblasts has also been previously shown in MVNT 39, 68, but more variable expression of nestin 39, 68 in line with our observations. We also noted intense expression of stem cell markers SOX2 and CD34, which has been consistently reported in CNS tumors, LEATS and FCD 35, 41, 57.

Immunohistochemistry for OTX1 showed striking labelling of VC, highlighting the subcortical extent of the MVNT. In murine cortex, homeobox gene *OTX1* is expressed in mid to late gestation during development primarily in early migrating neurones destined for deeper cortical layers V and VI 8 particularly in the temporal lobe 3. *OTX1* null mice show temporal cortical thinning 12 and spontaneous seizures 3. In the developing human brain, OTX1 is strongly expressed in the forebrain and proliferative layers of the neocortical precursors 30 as we also observed. In mature human cortex, OTX1 has been shown to be expressed in layer V pyramidal neurones 29, 38 as well as immature cells in FCD I and II 20, 29. The consistent expression of OTX1 in MVNT, appeared more pronounced than observed in ganglioglioma or the dysmorphic neurones of FCD 20 and could suggest that VC are derived from incomplete maturation or migration of progenitors destined for the deeper cortical layers. This could be one explanation for the predominant location of MVNT in deeper cortex compared to outer cortical layers. Focal TBR1 expression in VC is further support for their derivation from radial migrating progenitor cells or possibly subplate remnants 16.

### Glial differentiation and myelination

GFAP in MVNT highlighted a variable reactive glial component merging with an adjacent gliotic parenchyma, in keeping with previous observations 7, 24. However, using isoform GFAPδ we observed distinct populations of intra‐lesional cells as evidence for a glial‐cell component associated with MVNT. GFAPδ is normally restricted to glia residing in stem cell niches including the subventricular zone 65 as well as some malformations 29, 36 and astrocytic tumors 11 but with a less consistent expression in reactive gliosis 27. Furthermore, preferential labelling of smaller intralesional cells, rather than the VC, for pS6, PDGFRβ, PAX6 and nestin also argues for a mixed cellular composition in MVNT. Uniform labelling of both the VC and the smaller cells in MVNT for OLIG2 has been previously reported 7, 17, 24, 39, 68 and confirmed in our series. This transcription factor has a recognized oncogenic role in glioma growth 64, however in MVNT OLIG2 expression is noted in the context of overall reduced myelination and labelling of VC for myelin basic protein. One further hypothesis is that VC originate from OLIG2‐positive oligodendroglial progenitor cell types (OPC) 23 with developmentally arrested or partial maturation, resulting in defective myelination 9 and even aberrant neuronal differentiation 19. Indeed an occasionally observation in MVNT in this and previous reports 24 has been clustering of VC along vessels, reminiscent of developmental migration patterns of OPC 63. Recent description of a novel subtype of mild malformation of cortical development in frontal lobe epilepsy, associated with increased OLIG2 cells in the deep cortical layers and superficial white matter with myelin loss also draws comparison with MVNT 52.

### Neuroimaging, clinical presentation

Characteristic appearances reported in MRI of MVNT include multi‐nodularity 7, 68, satellite nodules away from a main lesion (Nagaishi, Yokoo, *et al*., 2015), hyperintensity on T2 and FLAIR and preferential involvement of the grey‐white matter junction following gyral contours, sometimes with a corrugated outer margin (Fukushima, Yoshida, *et al*., 2015). Combinations of these imaging features may enable the future distinction of MVNT pre‐operatively from other LEATs, gliomas and FCD. Interval MRI has also shown a lack of growth over 18 months 7, 24 as also supported by longer pre‐operative observational periods in this series.

From the 24 cases of MVNT reported 7, 17, 24, 68, the mean age at the time of surgery is 44.6 years. Although epilepsy was present in all our surgical samples it has been documented in just less than half of reported cases with an average age of onset of seizures of around 30 years. However, we include the youngest case, operated at 6 years confirming MVNT may be encountered in the pediatric age‐group. MVNT predominate in the temporal lobe with 18 of the 24 reported cases arising here, with a mesial component in eleven 7, 17, 24, 39, 68. ‘Ribbon‐like’ extensions of the tumour along the hippocampal formation and subiculum have been previously described, 68 and was striking in five of our cases which could potentially enhance hippocampal epileptogenicity, although presence of associated hippocampal sclerosis is variable.

### Epileptogensis and neurodegeneration

An older age at manifestation of epilepsy in MVNT compared to other epileptogenic lesions could be explained by their predominant subcortical location with limited cortical connectivity or relative functional inactivity. We explored acquisition of neurodegenerative changes in the VC for several reasons: accumulation of hyperphosphorylated tau/neurofibrillary tangle formation and p62 accumulation occurs early in dysmorphic neurones of FCD and ganglioglioma 26, 43, 54, indicating an enhanced vulnerability to degeneration. This could be an effect of mTOR over‐activity 43, that we demonstrated in MNVT with pS6 labelling, or increased neuronal excitability 67. By contrast in MVNT, although p62 was prominent, there was a striking absence of tangles or phosphorylated tau with AT8 immunohistochemistry. Indeed, even in the oldest post‐mortem case with high Braak stage AD, sparing of VC and nodules from tau accumulation was noted. Possible explanations include a functional disconnection of MVNT from the adjacent cortex preventing tau seeding 31 or indirectly it could reflect reduced excitability of VC if tau phosphorylation is activity driven.

We explored other potential pro‐epileptogenic cellular alterations in MVNT as previously investigated in related pathologies 2, 53, 58, 59 (Supporting Information Table S3). We identified some evidence for cation‐chloride cotransporter (KCC1/KCC2) imbalances in VC in keeping with cell immaturity 37, 49, reductions of inhibitory interneurons within lesions, immature astroglia 48, focal inflammation 14 as pro‐epileptogenic cellular alterations, that could operate in MVNT but warrant further in‐depth investigation.

In conclusion, MVNT are uncommon but distinct lesions mainly presenting in the temporal lobe in older patients with seizures. They appear indolent and lack any of the common mutations identified in LEATS or morphologically similar malformations. Our studies support a mixed cellular composition and expression of a range of immature markers which could indicate an origin from an aberrant progenitor cell type during development.

### Note added in proof

Since submission of this study, further cases and series have been reported, attesting to the indolent nature of MNVT 1, 4, 10, 40.

## Supporting information

Additional Supporting Information may be found in the online version of this article at the publisher's web‐site:

**Figure S1.** Comparative Labelling In Ganglioglioma Cases, TLE/Temporal Lobe In Temporal Lobe Epilepsy With Increased White Matter Neurones (Equivalent To Mild MCD/Malformation Of Cortical Development Type II) And Developmental Controls Of 13 Gestational Weeks. **A.** Tbr1 in gangliogliomas showed strong nuclear labelling in two cases as shown, but negative labelling in two further cases (not shown). **B.** OTX1 in ganglioglioma showed cytoplasmic positivity of dysmorphic neurons in a proportion of cases. **C.** SOX2 in gangliogliomas showed granular cytoplasmic (or peripheral cytoplasmic staining) of dysplastic ganglion cells which was variable between cases. **D.** OLIG2 was negative in the atypical neuronal cells of gangliogliomas or showed cytoplasmic granular staining but nuclear labelling was not a feature; (inset) PDGFRβ did not label the atypical ganglion cells. **E.** Calbindin showed strong labelling of the tumour and parenchyma with a sharp border with adjacent tissue and intense labelling of dysmorphic neuronal cells (inset). **F.** MCM2 labelling in gangliogliomas highlighted inflammatory cell component with variable nuclear positivity in a small proportion of the ganglion cells. **G.** TBR1 showed labelling of white matter neurons in mild MCD. **H.** There was no labelling of OTX1 in the white matter neurons with OTX1 in mild MCD and weak cytoplasmic labelling of small glial cells. **I.** In mild MCD, occasional weak cytoplasmic labelling of the single white matter neurons for SOX2 was noted. **J.** In mild MCD, the neuronal cells were not OLIG2 positive and only labelling of the small oligodendroglial cells seen. **K.** phosphor‐S6 labelling in mild MCD showed occasional labelling of the single white matter neurons and small glial cells and (**L**) KCC1 did not label the white matter neurons in Mild MCD. **M.** TBR2 in fetal cortex showed labelling of immature cells in the germinal matrix and in the periventricular zone and developing white matter. **N.** OTX1 in developmental controls showed a strong, predominantly peripheral ring of cytoplasmic labelling of the germinal matrix cells. **O.** With SOX2 strong labelling of primitive cells in the germinal matrix was seen. Bar = 50 microns (A–D,F,H–O); =100 microns in E and 200 microns on G (approximate based on original magnifications).

**Table S1.** Detail of the control cases used for comparative staining with the multinodular vacuolating neuronal tumour. These were used only for the markers where there is little available data in literature of labelling patterns. These controls tissues were selected from the University College London Epilepsy Society Brain and Tissue Bank. The staining patterns of control cases are shown in supplemental Figure 1 and is referred to in Supporting Information Table 2. TLE= temporal lobe epilepsy; MCD = malformation of cortical development

**Table S2.** Less frequent variants identified on NGS of eight cases of MNVT. 11 different polymorphism were identified involving 8 of the 33 genes tested.

**Table S3.** Comparison of growth patterns of multinodular vacuolating neuronal tumour (MNVT) and immunophenotypic characteristics of the atypical neuronal cells and vacuolated cells compared to other common cortical epilepsy pathologies in the main differential diagnosis: dysembryoplastic neuroepithelial tumour (DNT; classical form), ganglioglioma, focal cortical dysplasia (FCD IIB), mild malformation of cortical development type II (Mild MCD) and heterotopia. This is as based in reports in literature (as referenced in table), data reported in current study or ∼personal non‐ published observation. **In Bold** font the more potentially useful markers/tests to discriminate MNVT from other lesions in their differential diagnosis are highlighted. The diagnostic criteria for each lesion are based on WHO 2016 for tumours and ILAE for cortical malformations (44).
