# Supplementary figures and images for: Multinodular and vacuolating neuronal tumors in epilepsy: dysplasia or neoplasia?
Source: Brain Pathol. 2017 Sep 19;28(2):155–71. doi: 10.1111/bpa.12555 (PMC5887881; doi:10.1111/bpa.12555)

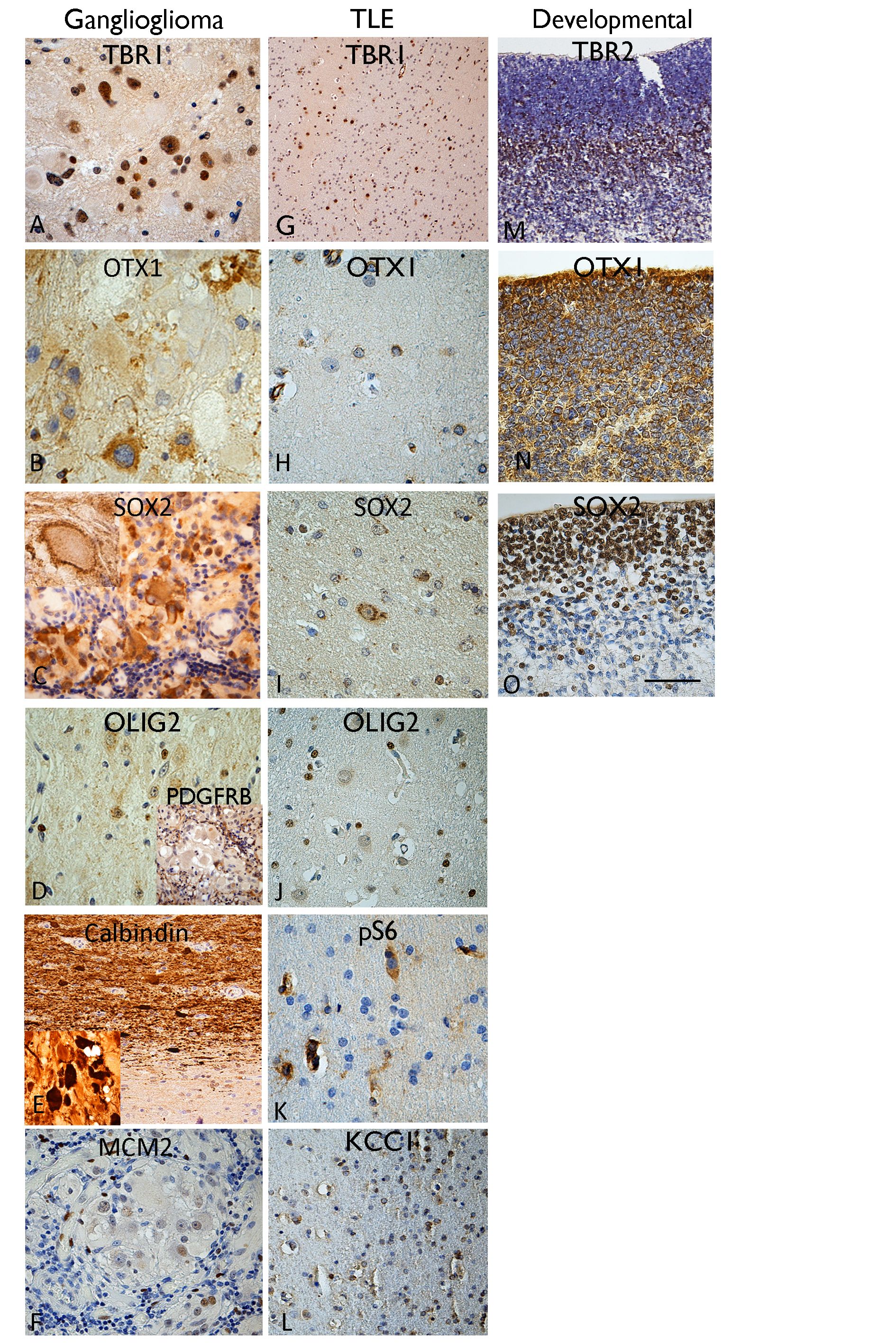

Supplement: Supplementary file 1 — Figure S1. Comparative Labelling In Ganglioglioma Cases, TLE/Temporal Lobe In Temporal Lobe Epilepsy With Increased White Matter Neurones (Equivalent To Mild MCD/Malformation Of Cortical Development Type II) And Developmental Controls Of 13 Gestational Weeks. A. Tbr1 in gangliogliomas showed strong nuclear labelling in two cases as shown, but negative labelling in two further cases (not shown). B. OTX1 in ganglioglioma showed cytoplasmic positivity of dysmorphic neurons in a proportion of cases. C. SOX2 in gangliogliomas showed granular cytoplasmic (or peripheral cytoplasmic staining) of dysplastic ganglion cells which was variable between cases. D. OLIG2 was negative in the atypical neuronal cells of gangliogliomas or showed cytoplasmic granular staining but nuclear labelling was not a feature; (inset) PDGFRβ did not label the atypical ganglion cells. E. Calbindin showed strong labelling of the tumour and parenchyma with a sharp border with adjacent tissue and intense labelling of dysmorphic neuronal cells (inset). F. MCM2 labelling in gangliogliomas highlighted inflammatory cell component with variable nuclear positivity in a small proportion of the ganglion cells. G. TBR1 showed labelling of white matter neurons in mild MCD. H. There was no labelling of OTX1 in the white matter neurons with OTX1 in mild MCD and weak cytoplasmic labelling of small glial cells. I. In mild MCD, occasional weak cytoplasmic labelling of the single white matter neurons for SOX2 was noted. J. In mild MCD, the neuronal cells were not OLIG2 positive and only labelling of the small oligodendroglial cells seen. K. phosphor‐S6 labelling in mild MCD showed occasional labelling of the single white matter neurons and small glial cells and (L) KCC1 did not label the white matter neurons in Mild MCD. M. TBR2 in fetal cortex showed labelling of immature cells in the germinal matrix and in the periventricular zone and developing white matter. N. OTX1 in developmental controls showed a strong, predo [file BPA-28-155-s003.jpg]
