# Supplementary material for: Multinodular and vacuolating neuronal tumors in epilepsy: dysplasia or neoplasia?
Source: Brain Pathol. 2017 Sep 19;28(2):155–71. doi: 10.1111/bpa.12555 (PMC5887881; doi:10.1111/bpa.12555)
Supplement: Supplementary file 2 — Table S1. Detail of the control cases used for comparative staining with the multinodular vacuolating neuronal tumour. These were used only for the markers where there is little available data in literature of labelling patterns. These controls tissues were selected from the University College London Epilepsy Society Brain and Tissue Bank. The staining patterns of control cases are shown in supplemental Figure 1 and is referred to in Supporting Information Table 2. TLE= temporal lobe epilepsy; MCD = malformation of cortical development [file BPA-28-155-s002.docx]

| Case | Diagnosis | Age at surgery/  Gender | Localisation | Nature of tissue | Outcome |
| --- | --- | --- | --- | --- | --- |
| 1 | Gangliocytoma (WHO Grade I) | 36/M | Lesion in right anterior temporal lobe | Lesionectomy | Lost to follow up |
| 2 | Ganglioglioma (WHO Grade I) | 39/M | Lesion in amygdala ; temporal lobe normal | Temporal lobectomy | Seizures continued following surgery |
| 3 | Ganglioglioma (WHO Grade I) | 55/F | Lesion in frontal lobe | Tumour resection | Lost to follow up |
| 4 | Ganglioglioma (WHO Grade I) | 20/M | Lesion in amygdala | Temporal lobectomy and hippocampectomy | Seizure free since surgery ; only auras |
| 5 | Temporal lobe in TLE. Excess white matter neurones/ mild MCD | 24/M | Temporal lobe.  MRI normal | Temporal lobectomy and hippocampectomy | Seizures continue post-operatively |

Supplemental Table 1. Detail of the control cases used for comparative staining with the multinodular vacuolating neuronal tumour. These were used only for the markers where there is little available data in literature of labelling patterns. These controls tissues were selected from the University College London Epilepsy Society Brain and Tissue Bank. The staining patterns of control cases are shown in supplemental Figure 1 and is referred to in supplemental table 2. TLE= temporal lobe epilepsy; MCD = malformation of cortical development
