# Supplementary material for: Multinodular and vacuolating neuronal tumors in epilepsy: dysplasia or neoplasia?
Source: Brain Pathol. 2017 Sep 19;28(2):155–71. doi: 10.1111/bpa.12555 (PMC5887881; doi:10.1111/bpa.12555)
Supplement: Supplementary file 3 — Table S2. Less frequent variants identified on NGS of eight cases of MNVT. 11 different polymorphism were identified involving 8 of the 33 genes tested. [file BPA-28-155-s001.docx]

| **Gene** | **Exon** | **Position**  **(hg19)/(hg38)** | **Nucleic change** | **AA change** | **Nucleic Name** | **SNP database no** | **Mutation effect** | **Total case**  **[case no]** |
| --- | --- | --- | --- | --- | --- | --- | --- | --- |
| PTCH1 | 12 | 63 (1665)  [chr9: 98238379]  [chr9: 95476097] | T🡪C | N🡪N (555) | c.1665T>C | rs1805155 | Silent  *With benign allele* | **3**  [1,9,8] |
| PTCH1 | 12 | 84 (1686)  [chr9: 98238358]  [chr9: 95476076] | C🡪T | A🡪A (562) | c.1686C>T | rs2066836  COSM1756288  COSM1756287 | Silent | **2**  [1,6] |
| IDH1 | 4 | 193 (315)  [chr2: 209113192]  [chr2: 208248468] | C🡪T | G🡪G (105) | c.315C>T | rs11554137  COSM1741220 | Silent | **2**  [2,6] |
| KDM6A | 20 | 173 (3111)  [chrX: 44938563]  [chrX: 45079318] | G🡪A | Q🡪Q (1037) | c.3111G>A | rs20539  COSM1179848 | Silent  *With benign allele* | **2**  [2,6] |
| SMARCA4 | 17 | -33  [chr19: 11129600]  [chr19: 11018924] | C🡪T | Intron | c.2439-33C>T | rs62129061 | Intron | **2**  [2,3] |
| HIST1H3C | 1 | 99 (99)  [chr6: 26045737]  [chr6: 26045509] | C🡪G | T🡪T (33) | c.99C>G | rs3752418  COSM3761735 | Silent | **1**  [5] |
| AKT3 | 2 | -43  [chr1: 243859061]  [chr1: 243695759] | G🡪C | Intron | c.47-43G>C | rs370256445 | Intron | **1**  [3] |
| PIK3CA | 21 | 300  [chr3: 178952181]  [chr3: 179234393] | T🡪C | 3’UTR | c.*29T>C | rs141178472 | 3’UTR | **1**  [6] |
| PIK3CA | 2 | +40  [chr3: 178917005]  [chr3: 179199217] | A -> G | Intron | c.352+40A>G | rs3729674 | Intron | 2  [3] |
| DEPDC5 | 16 | 14 (1095)  [chr22: 32200161]  [chr22: 31804175] | T -> C | D -> D (365) | c.1095T>C | rs79070552 | Silent | 1  [3] |
| DEPDC5 | 28 | -32  [chr22: 32239049] (hg19) | A -> G | Intron | c.2516-32A>G |  | Intron | 1  [6] |

Supplementary Table 2. Less frequent variants identified on NGS of eight cases of MNVT. 11 different polymorphism were identified involving 8 of the 33 genes tested.
