# Supplementary material for: Multinodular and vacuolating neuronal tumors in epilepsy: dysplasia or neoplasia?
Source: Brain Pathol. 2017 Sep 19;28(2):155–71. doi: 10.1111/bpa.12555 (PMC5887881; doi:10.1111/bpa.12555)
Supplement: Supplementary file 4 — Table S3. Comparison of growth patterns of multinodular vacuolating neuronal tumour (MNVT) and immunophenotypic characteristics of the atypical neuronal cells and vacuolated cells compared to other common cortical epilepsy pathologies in the main differential diagnosis: dysembryoplastic neuroepithelial tumour (DNT; classical form), ganglioglioma, focal cortical dysplasia (FCD IIB), mild malformation of cortical development type II (Mild MCD) and heterotopia. This is as based in reports in literature (as referenced in table), data reported in current study or ∼personal non‐ published observation. In Bold font the more potentially useful markers/tests to discriminate MNVT from other lesions in their differential diagnosis are highlighted. The diagnostic criteria for each lesion are based on WHO 2016 for tumours and ILAE for cortical malformations (44). [file BPA-28-155-s004.docx]

| **GROUPS** | | **EPILEPSY-ASSOCIATED TUMOURS FOCAL MCD DIFFUSE/GENERALISED MCD** | | | | | |
| --- | --- | --- | --- | --- | --- | --- | --- |
| **Feature** | | **DNT (classical)** | **GANGLIO**  **GLIOMA** | **MVNT** | **FCD II B** | **MILD MCD type II** | **HETEROTOPIA** |
| **Localisation** | | Cortex (any layers) + WM + leptomeninges  TL predilection[^1^](#_ENREF_1) | Cortex (anylayers) + WM + leptomeninges  TL predilection[^1^](#_ENREF_1) | WM ± deeper cortical layers V/VI  TL in 75% of reported cases | Cortex (all layers) + WM.  Any lobe ; More often extra-temporal/ favours frontal lobe | WM  TL predilection | WM ; periventricular or subcortical |
| **Neuronal morphology** | | Neurones in glioneuronal element not dysmorphic; normal laminar  distribution [^2^](#_ENREF_2)^,^ [^3^](#_ENREF_3). | DN/‘Dysplastic ganglion cells’  (can be bi-nucleate ;rarely vacuolated) | Vacuolated neurones predominant feature | DN + hypertrophic pyramidal cells | Normal appearing/mixed morphologies and interneurones | Mature appearing neurones and interneurons |
| **Lamination / organisation of neurones** | | Floating neurones can appear ‘entrapped’ | Aggregates of DN | Tend not to cluster  Occasional orientation of VC along blood vessels | Disordered lamination of cortex | Scattered single cells ; no organisation | Rudimentary laminae often evident[^4^](#_ENREF_4)^,^ [^5^](#_ENREF_5) |
| **Nodular versus diffuse architecture** | | Nodules ++ (complex form) | Nodular aggregates often present | Nodular > diffuse growth | Nodules not a feature | Diffuse involvement only | Either nodular or laminar |
| **Adjacent cortex** | | FCD IIIb variably reported | FCD IIIb variably reported | Normal lamination | Normal lamination | Normal lamination | Lamination may be abnormal eg PMG |
| Neuronal markers / Cortical layer markers | NeuN | +/- Tumour OLC [^3^](#_ENREF_3) | +/- (DN) | - (or weak) | ++ | + | +[^6^](#_ENREF_6) |
|  | **NFIL** | + Mature neurones  +/- Tumour OLC | ++ | + (present series NP only) | ++ (intense P and NP) | + (some cells) | + [^4^](#_ENREF_4) |
|  | **TBR1** | + Mature neurones  - Tumour OLC [^7^](#_ENREF_7) | +/- | ++/- (variable) | +/- [^7^](#_ENREF_7) | ↓[^8^](#_ENREF_8) | NR |
|  | TBR2 | - | - | - | + (BC)[^7^](#_ENREF_7) | NR | NR |
|  | MAP1b | + Mature neurones  - Tumour OLC [^3^](#_ENREF_3)^,^ [^7^](#_ENREF_7) | NR | + (intense) | +[^7^](#_ENREF_7) | - | NR |
| MyelinationOPC and OL | MBP | ↓ in WM[^1^](#_ENREF_1) | ↓ in WM[^1^](#_ENREF_1) | VC + (membranous)  ↓ myelin in nodular patches | ↓MBP in WM | Reduced myelin[^9^](#_ENREF_9) | NR |
|  | **OLIG2** | + Tumour cells [^2^](#_ENREF_2) | + (cytoplasmic) | ++ (in VC) | ↓OLIG2 cells[^10^](#_ENREF_10) | ↑ (OL)[^11^](#_ENREF_11) | NR |
|  | PDGFRα/β  NG2 | ↑PDGFβ~ | - | - | ↓NG2 and PDGFRα cells[^12^](#_ENREF_12) | WM neurones not positive with PDGFRβ | NR |
| Interneuronal markers | Cal-  bindin | +[^3^](#_ENREF_3) | ++in DN [^3^](#_ENREF_3) | - in VN | + (in cytomegalic interneurons)[^13^](#_ENREF_13) | ↓ cell numbers[^8^](#_ENREF_8) | Normal cells/abnormal distribution [^5^](#_ENREF_5)^,^ [^6^](#_ENREF_6) |
|  | NPY | NR | NR | - | DN +/-[^14^](#_ENREF_14) | NR | NR |
|  | KCC1 | NR | +[^15^](#_ENREF_15) | + | + [^15-17^](#_ENREF_15) | - | NR |
|  | KCC2 | NR | +[^15^](#_ENREF_15) | - | +[^15^](#_ENREF_15)  ↓ [^16^](#_ENREF_16) |  | NR |
| Developmental and  Stem cell | Nestin | + (frequent)[^3^](#_ENREF_3)^,^ [^18^](#_ENREF_18) | + [^19^](#_ENREF_19) | +/-[^20^](#_ENREF_20) | BC +/- [^21^](#_ENREF_21) |  | NR |
|  | **SOX2** | NR | ++ | +++ | +[^22^](#_ENREF_22) | +/- | NR |
|  | **OTX1** | - [^7^](#_ENREF_7) | +/- | +++ | DN+, BC +[^7^](#_ENREF_7)^,^ [^23^](#_ENREF_23) | - | NR |
|  | PAX6 | +/- Tumour cells[^3^](#_ENREF_3) | - | - | + (BC)[^23^](#_ENREF_23) | NR | NR |
|  | DCX | - Tumour cells[^3^](#_ENREF_3)^,^ [^24^](#_ENREF_24) | +[^24^](#_ENREF_24)  Low gene expression reported[^25^](#_ENREF_25) | +/-[^26^](#_ENREF_26) | DN , BC +[^24^](#_ENREF_24)^,^ [^27^](#_ENREF_27) | Not reported | -[^24^](#_ENREF_24) |
|  | Reelin | -[^3^](#_ENREF_3) | NR | - | - (DN/BC)[^14^](#_ENREF_14) |  | + I (small cells but no CRC in nodules)[^14^](#_ENREF_14)^,^ [^28^](#_ENREF_28) |
|  | CD34 | +/- | ++ | ++/+ | BC +/- | - | - |
|  | **Delta GFAP** | Focal +[^3^](#_ENREF_3) | - DN  Few glial cells[^29^](#_ENREF_29) | ++ | BC+[^29^](#_ENREF_29) | NR | No labelling [^29^](#_ENREF_29) |
| mTOR pathways | pS6 | +[^30^](#_ENREF_30) | ++[^30^](#_ENREF_30) | -/+ | ++[^31^](#_ENREF_31) | +/- | NR |
| Neuro-degenerative markers | AT8 | + (rare) | + (↑ with age)[^32^](#_ENREF_32) | - | + (DN; ↑ with age) [^33^](#_ENREF_33)^,^ [^34^](#_ENREF_34) | NR | NR |
|  | **P62** | NR | DN[^32^](#_ENREF_32) | ++ | BC /DN[^34^](#_ENREF_34) | NR | NR |
| Cell cycle markers | **MCM2** | NR | +/- | ++ | ++ (BC) | WM neurones negative | NR |
| **Molecular Genetic markers** | | FGFR1 mutations (58%)[^35^](#_ENREF_35)  BRAF mutations (0-50%)[^30^](#_ENREF_30)^,^ [^36^](#_ENREF_36) | BRAF mutation (39-56%)[^30^](#_ENREF_30)^,^ [^36^](#_ENREF_36) | point mutation MEK 1[^37^](#_ENREF_37)  BRAF mutation 0%[^37^](#_ENREF_37)^,^ [^38^](#_ENREF_38)  Recurring SNPs (DEPDC5, NPRL3, PIK3CA, SMO and TP53) | mTOR pathway mutations[^39^](#_ENREF_39)^,^ [^40^](#_ENREF_40)  (somatic and germline)  DEPDC5, NPRL3 [^41^](#_ENREF_41) and PIK3CA[^42^](#_ENREF_42) mutations | NR | DCX  FLNA  LIS1[^43^](#_ENREF_43)  (somatic and germline mutations) |

**Supplemental Table 3.** Comparison of growth patterns of multinodular vacuolating neuronal tumour (MNVT) and immunophenotypic characteristics of the atypical neuronal cells and vacuolated cells compared to other common cortical epilepsy pathologies in the main differential diagnosis : dysembryoplastic neuroepithelial tumour (DNT ; classical form), ganglioglioma, focal cortical dysplasia (FCD IIB) , mild malformation of cortical development type II (Mild MCD) and heterotopia. This is as based in reports in literature (as referenced in table), data reported in current study or ~personal non- published observation**. In Bold** font the more potentially useful markers/tests to discriminate MNVT from other lesions in their differential diagnosis are highlighted. The diagnostic criteria for each lesion are based on WHO 2016 for tumours and ILAE for cortical malformations[^44^](#_ENREF_44).

BC= balloon cells of FCD, CRC=Cajal Retzius cells, OL=oligodendroglial, OPC=OL progenitor cell, OLC = tumoural oligodendrocyte-like cell, MWt = molecular weight, PMG = polymicrogyria, P=phosphorylated (neurofilament), NP=non-phosphorylated (neurofilament), DN = dysmorphic neurones, DNT = Classical form of DNT; TL=temporal lobe, VC= vacuolated neuronal cell of MNVT, NR = no reports , WM = white matter.

1. Thom, M., Blumcke, I. & Aronica, E. Long-term epilepsy-associated tumors. *Brain Pathol* **22**, 350-79 (2012).

2. Komori, T. & Arai, N. Dysembryoplastic neuroepithelial tumor, a pure glial tumor? Immunohistochemical and morphometric studies. *Neuropathology* **33**, 459-68 (2013).

3. Thom, M. et al. One hundred and one dysembryoplastic neuroepithelial tumors: an adult epilepsy series with immunohistochemical, molecular genetic, and clinical correlations and a review of the literature. *J Neuropathol Exp Neurol* **70**, 859-78 (2011).

4. Garbelli, R. et al. Layer-specific genes reveal a rudimentary laminar pattern in human nodular heterotopia. *Neurology* **73**, 746-53 (2009).

5. Thom, M., Martinian, L., Parnavelas, J.G. & Sisodiya, S.M. Distribution of cortical interneurons in grey matter heterotopia in patients with epilepsy. *Epilepsia* **45**, 916-23 (2004).

6. Meroni, A. et al. Nodular heterotopia: a neuropathological study of 24 patients undergoing surgery for drug-resistant epilepsy. *Epilepsia* **50**, 116-24 (2009).

7. Hadjivassiliou, G. et al. The application of cortical layer markers in the evaluation of cortical dysplasias in epilepsy. *Acta Neuropathol* **120**, 517-28 (2010).

8. Richter, Z. et al. Characterization of neurons in the cortical white matter in human temporal lobe epilepsy. *Neuroscience* **333**, 140-50 (2016).

9. Garbelli, R. et al. Blurring in patients with temporal lobe epilepsy: clinical, high-field imaging and ultrastructural study. *Brain* **135**, 2337-49 (2012).

10. Muhlebner, A. et al. Neuropathologic measurements in focal cortical dysplasias: validation of the ILAE 2011 classification system and diagnostic implications for MRI. *Acta Neuropathol* **123**, 259-72 (2012).

11. Schurr, J. et al. Mild malformation of cortical development with oligodendroglial hyperplasia in frontal lobe epilepsy: A new clinico-pathological entity. *Brain Pathol* (2016).

12. Shepherd, C. et al. A quantitative study of white matter hypomyelination and oligodendroglial maturation in focal cortical dysplasia type II. *Epilepsia* **54**, 898-908 (2013).

13. Andre, V.M. et al. Cytomegalic interneurons: a new abnormal cell type in severe pediatric cortical dysplasia. *J Neuropathol Exp Neurol* **66**, 491-504 (2007).

14. Thom, M. et al. Cajal-Retzius cells, inhibitory interneuronal populations and neuropeptide Y expression in focal cortical dysplasia and microdysgenesis. *Acta Neuropathol* **105**, 561-9 (2003).

15. Aronica, E. et al. Differential expression patterns of chloride transporters, Na+-K+-2Cl--cotransporter and K+-Cl--cotransporter, in epilepsy-associated malformations of cortical development. *Neuroscience* **145**, 185-96 (2007).

16. Talos, D.M. et al. Altered inhibition in tuberous sclerosis and type IIb cortical dysplasia. *Ann Neurol* **71**, 539-51 (2012).

17. Sen, A. et al. Increased NKCC1 expression in refractory human epilepsy. *Epilepsy Res* **74**, 220-7 (2007).

18. Sung, C.O. & Suh, Y.L. Different pattern of expression of nestin in the non-specific form of dysembryoplastic neuroepithelial tumors compared to the simple and complex forms. *J Neurooncol* **92**, 7-13 (2009).

19. Duggal, N. & Hammond, R.R. Nestin expression in ganglioglioma. *Exp Neurol* **174**, 89-95 (2002).

20. Yamaguchi, M. et al. Multinodular and vacuolating neuronal tumor affecting amygdala and hippocampus: A quasi-tumor? *Pathol Int* **66**, 34-41 (2016).

21. Han, C.W. et al. Immunohistochemical analysis of developmental neural antigen expression in the balloon cells of focal cortical dysplasia. *J Clin Neurosci* **18**, 114-8 (2011).

22. Orlova, K.A. et al. Early progenitor cell marker expression distinguishes type II from type I focal cortical dysplasias. *J Neuropathol Exp Neurol* **69**, 850-63 (2010).

23. Lamparello, P. et al. Developmental lineage of cell types in cortical dysplasia with balloon cells. *Brain* **130**, 2267-76 (2007).

24. Srikandarajah, N. et al. Doublecortin expression in focal cortical dysplasia in epilepsy. *Epilepsia* **50**, 2619-28 (2009).

25. Becker, A.J. et al. Mutational and expression analysis of the reelin pathway components CDK5 and doublecortin in gangliogliomas. *Acta Neuropathol* **104**, 403-8 (2002).

26. Nagaishi, M. et al. Localized overexpression of alpha-internexin within nodules in multinodular and vacuolating neuronal tumors. *Neuropathology* **35**, 561-8 (2015).

27. Mizuguchi, M., Yamanouchi, H., Becker, L.E., Itoh, M. & Takashima, S. Doublecortin immunoreactivity in giant cells of tuberous sclerosis and focal cortical dysplasia. *Acta Neuropathol* **104**, 418-24 (2002).

28. Rossini, L., Tassi, L., Spreafico, R. & Garbelli, R. Heterotopic reelin in human nodular heterotopia: a neuropathological study. *Epileptic Disord* **14**, 398-402 (2012).

29. Martinian, L. et al. Expression patterns of glial fibrillary acidic protein (GFAP)-delta in epilepsy-associated lesional pathologies. *Neuropathol Appl Neurobiol* **35**, 394-405 (2009).

30. Prabowo, A.S. et al. BRAF V600E mutation is associated with mTOR signaling activation in glioneuronal tumors. *Brain Pathol* **24**, 52-66 (2014).

31. Miyata, H., Chiang, A.C. & Vinters, H.V. Insulin signaling pathways in cortical dysplasia and TSC-tubers: tissue microarray analysis. *Ann Neurol* **56**, 510-9 (2004).

32. Prabowo, A.S. et al. Expression of neurodegenerative disease-related proteins and caspase-3 in glioneuronal tumours. *Neuropathol Appl Neurobiol* **41**, e1-e15 (2015).

33. Sen, A. et al. Pathological tau tangles localize to focal cortical dysplasia in older patients. *Epilepsia* **48**, 1447-54 (2007).

34. Iyer, A. et al. Cell injury and premature neurodegeneration in focal malformations of cortical development. *Brain Pathol* **24**, 1-17 (2014).

35. Rivera, B. et al. Germline and somatic FGFR1 abnormalities in dysembryoplastic neuroepithelial tumors. *Acta Neuropathol* **131**, 847-63 (2016).

36. Qaddoumi, I. et al. Genetic alterations in uncommon low-grade neuroepithelial tumors: BRAF, FGFR1, and MYB mutations occur at high frequency and align with morphology. *Acta Neuropathol* **131**, 833-45 (2016).

37. Huse, J.T. et al. Multinodular and vacuolating neuronal tumors of the cerebrum: 10 cases of a distinctive seizure-associated lesion. *Brain Pathol* **23**, 515-24 (2013).

38. Bodi, I. et al. Two cases of multinodular and vacuolating neuronal tumour. *Acta Neuropathol Commun* **2**, 7 (2014).

39. Lim, J.S. & Lee, J.H. Brain somatic mutations in MTOR leading to focal cortical dysplasia. *BMB Rep* **49**, 71-2 (2016).

40. Nakashima, M. et al. Somatic Mutations in the MTOR gene cause focal cortical dysplasia type IIb. *Ann Neurol* **78**, 375-86 (2015).

41. Ricos, M.G. et al. Mutations in the mammalian target of rapamycin pathway regulators NPRL2 and NPRL3 cause focal epilepsy. *Ann Neurol* **79**, 120-31 (2016).

42. Jansen, L.A. et al. PI3K/AKT pathway mutations cause a spectrum of brain malformations from megalencephaly to focal cortical dysplasia. *Brain* **138**, 1613-28 (2015).

43. Jamuar, S.S. et al. Somatic mutations in cerebral cortical malformations. *N Engl J Med* **371**, 733-43 (2014).

44. Blumcke, I. et al. The clinicopathologic spectrum of focal cortical dysplasias: a consensus classification proposed by an ad hoc Task Force of the ILAE Diagnostic Methods Commission. *Epilepsia* **52**, 158-74 (2011).
